# Analysis of Thermal Aging Influence on Selected Physical and Mechanical Characteristics of Polyaddition and Polycondensation Poly(dimethylsiloxane)

**DOI:** 10.3390/polym15193857

**Published:** 2023-09-22

**Authors:** Ewelina Chmielnicka, Małgorzata Szymiczek, Sara Sarraj, Sebastian Jurczyk

**Affiliations:** 1 Łukasiewicz Research Network—Institute of Engineering of Polymer Materials and Dyes, M. Skłodowskiej-Curie St. 55, 87-100 Torun, Poland; sebastian.jurczyk@impib.lukasiewicz.gov.pl; 2 Institute of Theoretical and Applied Mechanics, Faculty of Mechanical Engineering, Silesian University of Technology, Konarskiego St. 18A, 44-100 Gliwice, Poland; malgorzata.szymiczek@polsl.pl (M.S.); sara.sarraj@polsl.pl (S.S.)

**Keywords:** tensile strength, dynamic mechanical analysis, spectral analysis

## Abstract

The aim of the study was to determine the effect of accelerated thermal aging on the properties of selected poly(dimethylsiloxanes) (PDMS) differing in viscosity and hardness. This was related to the potential application for specialist casting molds with complex geometry. Four polyaddition silicones and two polycondensation ones were selected. As part of the work, tensile strength, hardness, density, roughness, and Dynamic Mechanical Analysis (DMA) and Fourier Transform Infrared Spectroscopy (FTIR) were tested, which allowed us to determine the degree of degradation of the analyzed materials subjected to thermal aging at a temperature of 150 ± 2 °C. The aging temperature was conditioned by the parameters of the materials that can be cast into molds made of poly(dimethylsiloxanes) e.g., with polymer resins, for which the exothermic peak ranges from 100 to 200 °C depending on the volume. It was observed that the initial Shore A hardness value affects parameters such as tensile strength or the amount of value change (its increase or decrease) after thermal aging. It can also be concluded that for polyaddition PDMS, the viscosity of the material has an effect on the size of the relative elongation value after thermal aging.

## 1. Introduction

Due to their functional characteristics and relatively easy modification, organosilicon polymers are widely used in many industrial fields, such as electronics, automotive, construction, and food. Depending on the chemical structure, polysilanes (additional silicon atom), polycarboxylates (carbon atom), polysiloxanes (oxygen atom), polysilazanes (nitrogen bonded to hydrogen or instead of hydrogen a hydrocarbon group), polyborosilanes (boron atom), or polysilicocarbodiimides are distinguished [1]. By using different substituents, thermal resistance, electrical or optical properties, and also solubility can be modified [1]. 

Polysiloxanes contain Si-O-Si bonding in their structure; they are most often obtained through the polymerization of cyclic siloxanes with ring opening. The monomer for this process is prepared in the “direct process”. They contain difunctional units consisting of groups (R), which are hydrogen or organic groups, and terminal units that are most often a monofunctional siloxyl unit [2]. Poly(dimethylsiloxanes) are synthesized through the hydrolysis of dichlorodimethylsilane. The main degradation products in a vacuum are the tetramer and trimer [3]. The behavior of the material during aging is influenced by the method of obtaining poly(dimethylsiloxane). Materials obtained in the polycondensation reaction have shorter chains and low molecular weight, and the molecular weight and polydispersity can also be controlled in a limited way. The opposites are polyaddition PDMS, characterized by the absence of a by-product, greater chain length control, and macromolecule structure. The mechanism of polymerization ring opening of the most chain cyclosiloxane and polycondensation of polymer chains proposed in the paper allows us to increase the molecular weight, which conditions the properties, such as strength. Substrates, structure, and molecular weight significantly affect the operational characteristics of materials. Additionally, functional properties can be modified with the use of new crosslinking systems [3]; fillers changing, for example, thermal conductivity [4,5,6,7]; electrical properties [7]; and flammability [8], which allow for the control of important characteristics in the field of specific applications. Polysiloxanes are hydrophobic, chemically neutral, and characterized by low viscosity and good thermal stability, which have a significant impact on work efficiency and durability during operation. This means that they are capable of operating under extreme conditions without a negative impact on their physical and mechanical properties, which is particularly important in applications requiring materials with high heat resistance, such as electronic devices. 

Thermal stability is also significant from an economic point of view, as it extends the lifespan of components, and thus devices, reducing the need for their frequent replacement. This means that organosilicon materials with high thermal stability can contribute to reducing operational and maintenance costs. Additionally, these types of materials are known for their resistance to various environmental factors, such as UV radiation, moisture, and various chemical compounds. This resistance, combined with thermal stability, makes them materials for applications in extreme environmental conditions where other materials may not be effective. Determining the impact of processes such as thermal aging on organosilicon materials is crucial for optimizing their lifespan. Therefore, research and development in this field are essential for the continuous improvement of these materials and finding new, innovative applications. 

The resistance to high temperatures and other properties of organosilicon materials are extremely important for their effective use in many industrial and scientific applications. Maintaining these properties during long-term operation is crucial for efficiency, durability, and cost reduction related to the use of these materials. The thermal degradation of organosilicon materials is a complex process that involves a series of chemical reactions. Essentially, under the influence of high temperature, silicones may undergo processes such as oxidation, hydrolysis, and polymer chain cracking, which can lead to a loss of their original properties [9,10,11,12,13]. The mechanisms of degradation of organosilicon materials under the influence of high temperature are complex and diverse, but they have significant implications for the durability of these materials. Therefore, understanding these processes is key to optimizing the use of organosilicon polymers. However, it is difficult to find differences in operational characteristics between polyaddition PDMS and polycondensation PDMS.

In the available literature, there are few publications presenting research results on the aging resistance of condensation-cured and addition-cured PDMS. Given the unique properties of silicones, which make them applicable in many areas, it is purposeful to deepen the understanding of the impact of operation in adverse working conditions, such as high temperature, and its influence on material changes. These changes can significantly affect the durability of products made from these materials and, therefore, their reliability and lower long-term operating costs. For this reason, this publication focuses on presenting the impact of accelerated thermal aging on the key characteristics of two types of PDMS.

## 2. Research

The aim of the research was to assess the impact of thermal aging on the characteristics of additive and condensation polydimethylsiloxanes, differentiated in terms of viscosity and hardness. The evaluation was made based on changes in density, hardness, tensile stress and tensile strain, measurement of the storage modulus and loss modulus, and spectral analysis.

## 3. Materials and Methods

The research was conducted on casting polydimethylsiloxanes:

- addition type: RTV 3428 (marked AA) by Elkem (Warsaw, Poland), ZA 28 (AB) (Zhermack Industrial (Warsaw, Poland)) and HT 33 (AC) (Zhermack Industrial (Warsaw, Poland)), RTV 3040SB (AD) (Elkem (Warsaw, Poland))
- condensation type: RTV 3318 (KE) (Elkem (Warsaw, Poland)), RTV 3325 (KF) (Elkem (Warsaw, Poland)), which can be used as molding materials for parts with complex geometry.

The materials were selected with heuristic methods taking into account hardness and viscosity, which are important for technological processes and application uses. All materials were available in a two-component form. The components were mixed in the proportions provided in Table 1. The properties are presented in Table 1.

**Figure 1:**
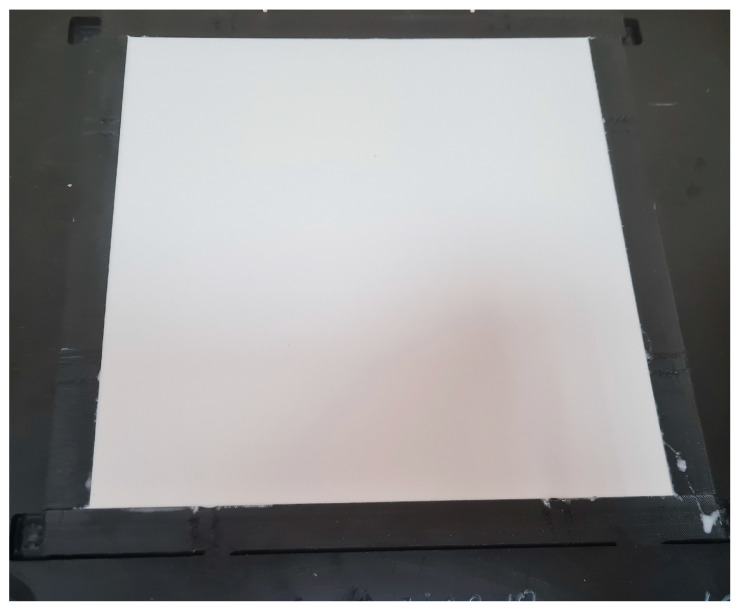
Sample KF after being cast into the mold.

Materials for the study were prepared in laboratory conditions (temperature 21.3 ÷ 22.6 °C, RH = 35 ÷ 48%) in accordance with the manufacturer’s recommendations in terms of the mass content of the silicone base and catalyst (Table 1). The materials were mixed using a High-Speed Dissolver Dispermat LC30 mixer (VMA-Getzmann GMBH, Reichshof, Germany) at a speed of 2000 rpm for about 5 min. The intensive homogenization process led to significant aeration of the materials. To limit the potential negative impact of structural discontinuities in their cross-section before casting the samples, they were subjected to deaeration under a vacuum of 0.06 MPa for a time that was dependent on the viscosity of the materials (Table 1); then, they were cast into polyethylene molds measuring 200 × 200 mm (Figure 1). The thickness of the obtained samples was 4 mm. The prepared materials were conditioned at a temperature of 60 ± 2°C for a time of 1 h, and then, samples were cut according to the appropriate standards.

### 3.1. Density

**Figure 2:**
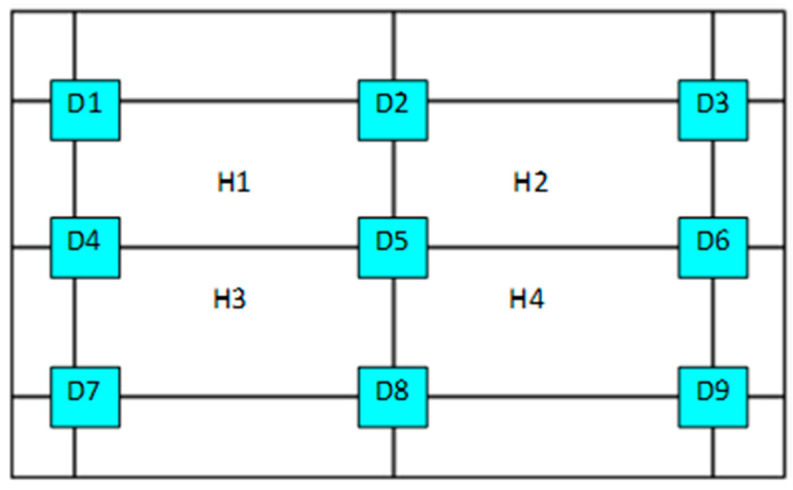
View of the sample collection areas.

The density of the materials was determined by the immersion method according to the standard PN-EN ISO 1183-1:2019-05 [21] using an analytical scale with a density measurement attachment Mettler-Toledo XS105 DualRange (Mettler-Toledo GmbH, Schwerzenbach, Switzerland). The test was conducted in distilled water at a temperature of 25 ± 0.5 °C. The tests were carried out on 9 samples of dimensions 10 × 10 mm (marked D1–D9), taken from different areas of the cast plates (intersection lines) according to Figure 2. Each sample was tested three times. 

### 3.2. Hardness 

The hardness of the materials was determined using the Shore A method in accordance with the PN-EN ISO 868:2005 standard [22] using a Zwick&Roell Shore A durometer (Zwick GmbH & Co., Ulm, Germany). Ten hardness measurements were performed on each sample measuring 100 × 100 mm (marked H1–H4 according to Figure 2). Before testing, the samples were conditioned for 24 h at a temperature of 25 ± 2 °C and a relative humidity of 50 ± 2%. The study was conducted under the conditioning conditions.

### 3.3. Mechanical Testing 

Tensile strength tests were carried out on five type 5A specimens in accordance with the PN-EN ISO 527-2 standard [20]. The samples were prepared by cutting, ensuring the continuity of the structure (no notches) from areas marked H1–H4, which allowed for the elimination of the effects of edge conditions. The tests were carried out on a LaborTech LabTest 6.100 strength testing machine (LaborTech, Opava, Czech Republic) equipped with mechanical grips ensuring stable holding of samples. The characteristics tested, i.e., tensile stress at break and elongation at break, were determined using a LaborTech ONE2 video extensometer (LaborTech, Opava, Czech Republic) in accordance with PN-EN ISO 527-1 [23] and PN-EN ISO 527-2. The elongation speed was 100 mm/min. Before testing, the samples were conditioned for 24 h under the test conditions. Tests were carried out in an air-conditioned room with an ambient temperature of 25 ± 2 °C and humidity of 50 ± 2%.

### 3.4. Roughness 

Height roughness parameters (Rz—height of irregularities) were determined on the tested samples using a TR200 profilometer (TIME Instruments, Beijing, China). Ten separate roughness measurements were made on each sample. Before testing, the samples were conditioned for 24 h at a temperature of 25 ± 2 °C and 50 ± 2% RH (Relative Humidity).

### 3.5. Dynamic Mechanical Analysis (DMA) 

The tests were carried out on a DMA/SDTA 861e Mettler-Toledo device (Mettler-Toledo GmbH, Schwerzenbach, Switzerland) on rectangular samples. The measurement was performed in shear mode at a temperature from −150 to 100 ± 0.5 °C, the deformation force was 14 N, and the frequency was 1 Hz. The study was conducted on a single sample for each of the materials. The samples for testing were in the shape of cylinders with a diameter of 4 mm and a height of 2 ± 0.05 mm. The storage modulus G′ and the loss modulus G″ were determined for samples before and after thermal aging.

### 3.6. Fourier Transform Infrared Spectroscopy (FTIR) 

FTIR spectra were obtained in attenuated total reflection (ATR) mode using an IRSpirit FTIR spectrophotometer (Shimadzu Corporation, Kyoto, Japan). Spectra were collected over the mid-infrared range (4000–600 cm^−1^) with a resolution of 4 cm^−1^ and 20 scans. Prior to testing, background spectrum was collected. After each test the ATR diamond crystal was cleaned using ethanol. The tests were carried out on reference (not aged) and thermally aged samples. 

### 3.7. Accelerated Thermal Aging 

**Figure 3:**
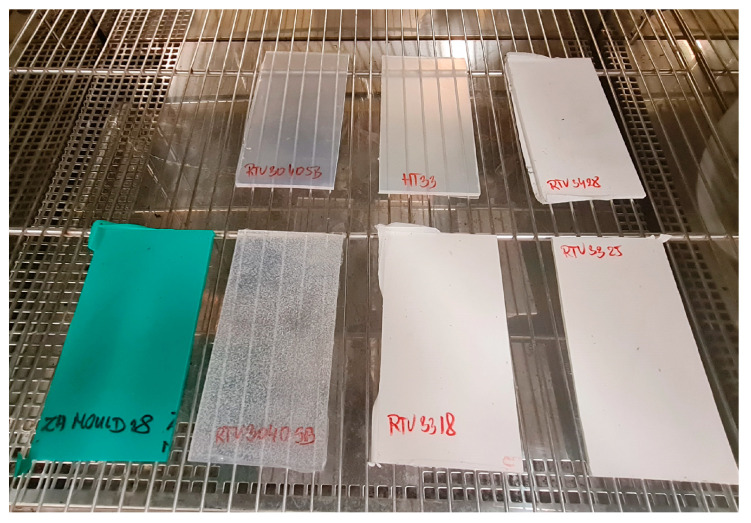
Samples subjected to the thermal aging process in a climatic chamber.

The cast PDMS plates underwent an accelerated aging process in a DY 600 CE S ATT climatic chamber (Angelantoni Test Technologies S.r.l., Perugia, Italy) at a temperature of 150 ± 2 °C for 384 h. This is a time corresponding to 100 to 500 moldings, depending on the volume, temperature, hardener, and release agent adapted to the material [24]. The aging temperature was conditioned by the processing parameters of the materials, which may potentially be formed in them (for example, polymer resins, for which the exothermic peak ranges from 100 to 200 °C depending on the volume) [25]. Figure 3 shows the view of the research material during thermal aging. The evaluation of characteristic changes was based on the measurement of tensile stress at break, strain at break, hardness, roughness, storage modulus and loss modulus (DMA), density, and FTIR spectral analysis. The samples for testing were collected according to Figure 2. To assess changes resulting from aging, the results obtained were compared to those of samples not subjected to the accelerated thermal aging process (reference samples).

## 4. Results

### 4.1. Density 

Figure 3 presents the average values of the density measurement conducted for samples before and after aging. The results shown are the average density values obtained for each material. The error bars correspond to the standard deviation of the received measurements as a measure of heterogeneity.

**Figure 4:**
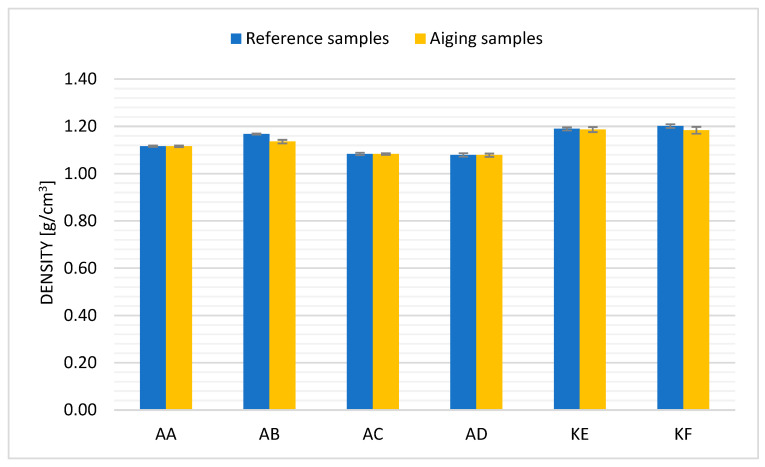
Density for samples before and after thermal aging.

As observed (Figure 4), silicones, regardless of their type—polyadditive and polycondensation, show a decrease in material density after thermal aging. The largest percentage change in value compared to the reference sample was observed for sample AB (2.7%) and KF (1.49%). Changes in density values are slight and within the error margin. The lack of changes in the density of the tested samples indicates a marginal impact of the aging process on the density arrangement of polymer chains in the material’s structure.

### 4.2. Hardness 

Figure 4 presents the average Shore A hardness values obtained for samples before and after thermal aging. The results shown are the average values from 10 measurements with error bars referring to the standard deviation of the received values.

**Figure 5:**
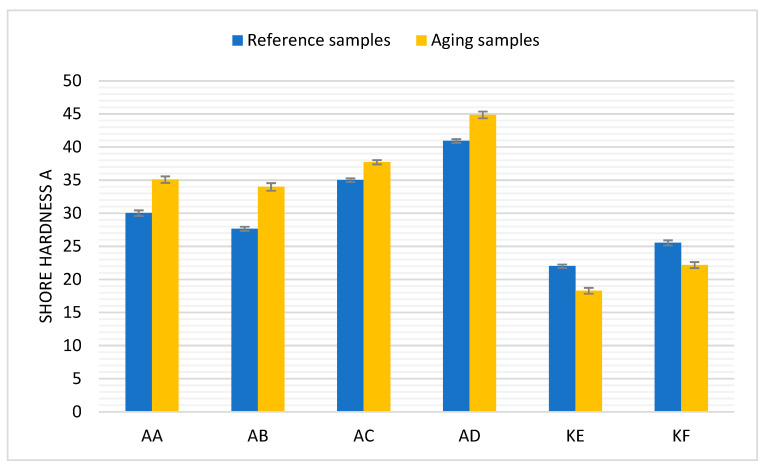
Shore A hardness for samples before and after thermal aging.

As observed (Figure 5), polyadditive silicones show a tendency to increase their hardness while polycondensation silicones after aging are characterized by lower hardness compared to the reference material. The largest increase in hardness was observed for the AB sample, which was over 22%, while the smallest was observed for the AC sample, which was just above 7%. It can be stated that temperature affects the change in hardness depending on the synthesis reaction. The increase in hardness for addition-cure PDMS is most likely due to further crosslinking of the material, initiated by the thermal energy supplied during the aging process. On the other hand, the decrease in hardness for condensation-cure PDMS may be due to degradation and the relaxation of internal stresses.

### 4.3. Mechanical Testing

In the Supplementary Materials, there is the example force—relative elongation curves before (Figures S1a and S2a, Supplementary Materials) and after aging (Figures S1b and S2b, Supplementary Materials), respectively, for polyadditive PDMS (AB) and polycondensation PDMS (KE). Analyses were carried out for repeatable forces. It was assumed that permissible force differences should not exceed 15% compared to the average value. Differences were primarily due to the properties of the material and the method of sample cutting.

**Figure 6:**
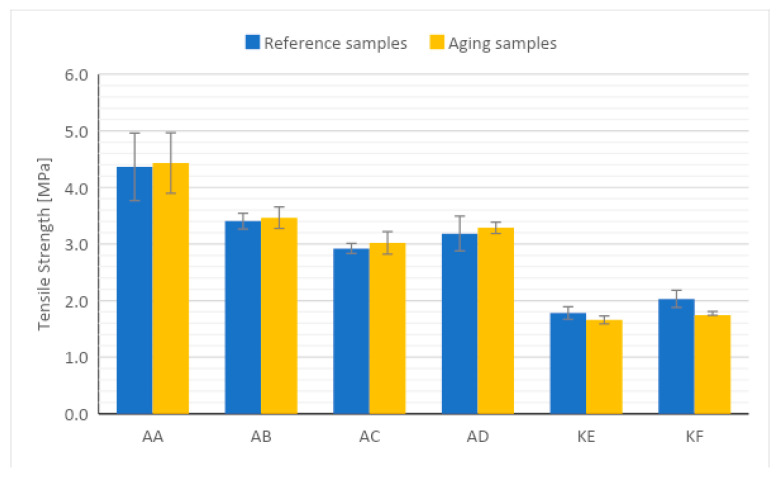
Tensile strength results.

**Figure 7:**
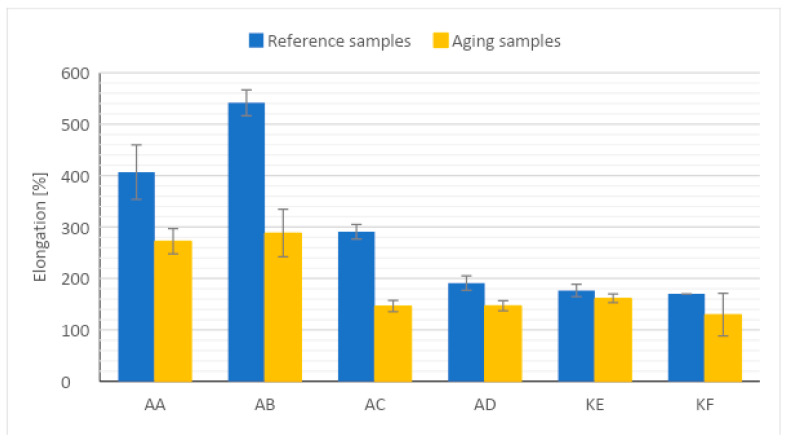
Relative elongation results.

The graphs of changes in the tensile strength before and after aging are presented in Figure 6, and the elongation at break is presented in Figure 7.

After aging for polyaddition PDMS (Figure S1b, Supplementary Materials), higher breaking forces, smaller relative elongations, but a greater scatter of results are observed, which may indicate the strengthening and stabilization of the material. There are no breaks in the curves, as in Figure S1a, Supplementary Materials), so it can be assumed that a crosslinking process occurred during aging. Such a situation was observed for AB and AC.

In the case of condensation PDMS (Figure S2, Supplementary Materials), no curve breaks were observed in any instance as in Figure S1a (Supplementary Materials), but there is a reduction in the tear strength and the relative elongation. This is due to the presence of shorter chains in the material, which after thermal aging show weakening and a tendency to tear.

As a result of conducting a static tensile test and determining the parameters of tensile strength and relative elongation of the tested materials, an increase in tensile strength of polyadditive silicones was observed. The change in the average value of tensile strength was 3.4% for AC, 3.2% for AD, 1.5% for AA, and 1.7% for AB compared to materials not subjected to the action of high temperature. Condensation silicones showed a decrease in tensile strength, where the average differences in the decrease in value were 6.9% for KE and 14.3% for KF. Based on the obtained results, significant changes in the value of relative elongation can be noticed. The tested materials after thermal aging showed a decrease in the value of relative elongation regardless of the type of material tested. The highest difference in the value of relative elongation decreased by about 50% for the AC silicone sample. Changes in the other additive silicones AB, AD and AA were in the range of 23.1 to 46.7% change in elongation value. Condensation silicone showed a smaller decrease in relative elongation after thermal aging. The difference in KE silicone was 8.6%. On the curves (Figure S1b) from tensile tests, you can also observe the uneven behavior of the samples during the test. Greater variation in the elongation of the same series of measurements was observed. The increase in the average tensile strength value for polyadditive silicones is identical to the results obtained from the Shore hardness measurement, where an increase in hardness was also observed. The increase in value is likely related to the structural construction of polyadditive PDMS, which consists of long chains. Exposure of materials to high temperature contributed to the crosslinking of the structure, which as a result exhibits better strength properties, but a smaller relative elongation. For condensation PDMS KE and KF, the tensile strength value probably dropped due to short chains in the material structure, which underwent degradation and the breaking of some bonds due to the influence of high temperature. 

The results of the tensile strength tests are consistent with the Shore A hardness test results. In samples where an increase in hardness was observed, there was a lower value of elongation at the break, indicating reduced material elasticity. At the same time, for condensation PDMS, where a decrease in hardness was observed, a decrease in elongation at the break may indicate material degradation. As a result of degradation, the polymer chains were likely shortened, and their possible deformation accounts for the value of this parameter. In the case of reduced polymer chain length (molecular weight), the material is not capable of as significant a deformation as in the case of long polymer chains.

### 4.4. Roughness

**Figure 8:**
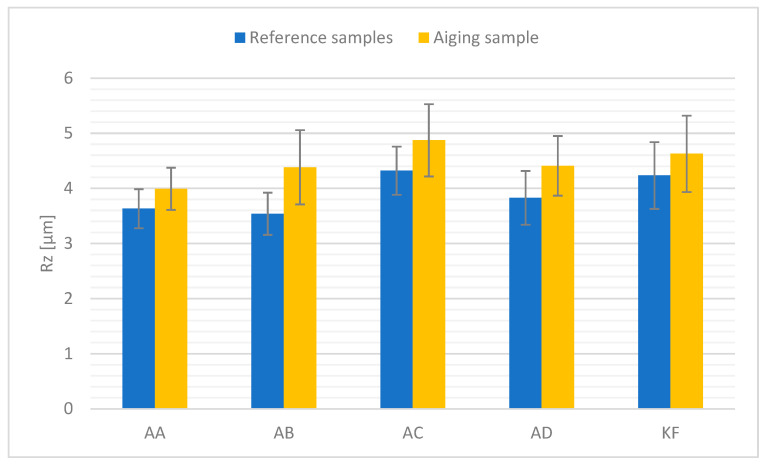
Surface roughness measurement results.

Figure 8 presents the values of roughness Rz (height of irregularities) measurements of the surfaces of the tested materials.

Based on the surface examination of the test material, a change in surface roughness was observed for all tested samples. The test material subjected to high temperature shows a higher surface roughness compared to the reference samples. The average value of changes was recorded within 9–24% in relation to the reference material. The largest change is noticeable for the polyadditive silicone AB, amounting to 23.8%, while the smallest change was observed for the condensation silicone KF—9.3%. Condensation silicone KE was not placed on Figure 8 because the low hardness of the material made it impossible to measure the roughness. In the case of all the samples studied, the observed changes are likely due to the reorganization of the material’s structure, which resulted in the displacement of polymer chains within the surface of the samples. It is also possible that some surface degradation of the material occurred due to the action of atmospheric oxygen at elevated temperatures.

### 4.5. Dynamic Mechanical Analysis (DMA) 

**Figure 9:**
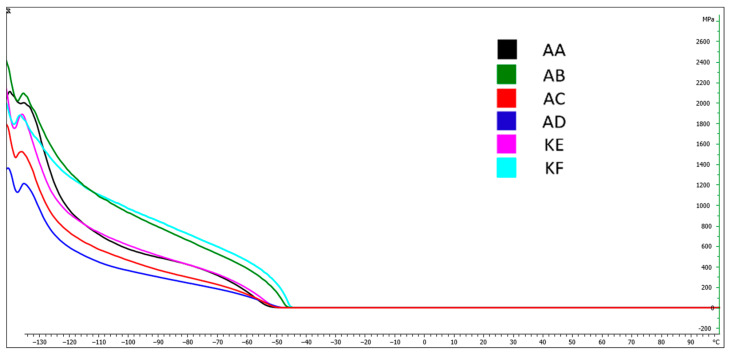
DMA curves−Storage modulus G′ for reference samples.

**Figure 10:**
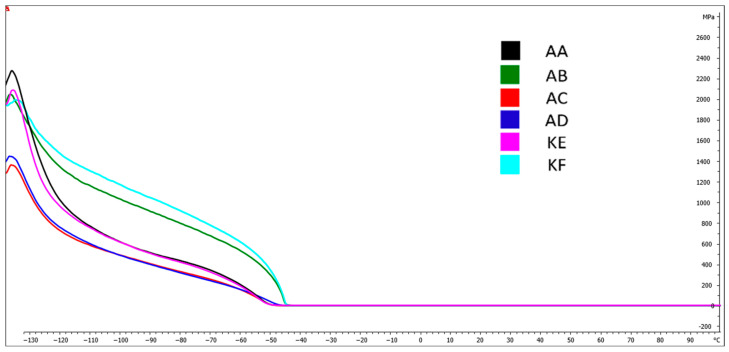
DMA curves−Loss modulus G′ for thermally aged samples.

**Figure 11:**
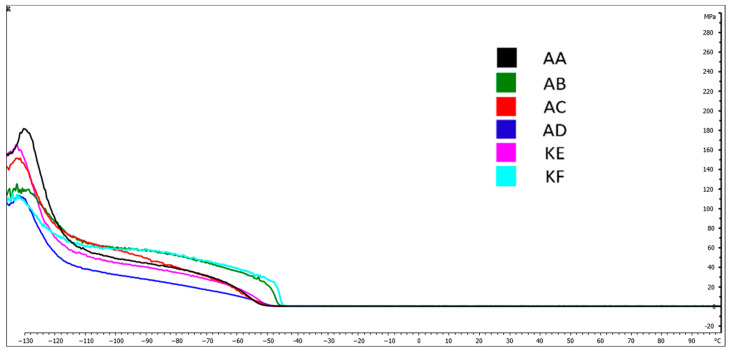
DMA curves−Loss modulus G″ for reference samples.

**Figure 12:**
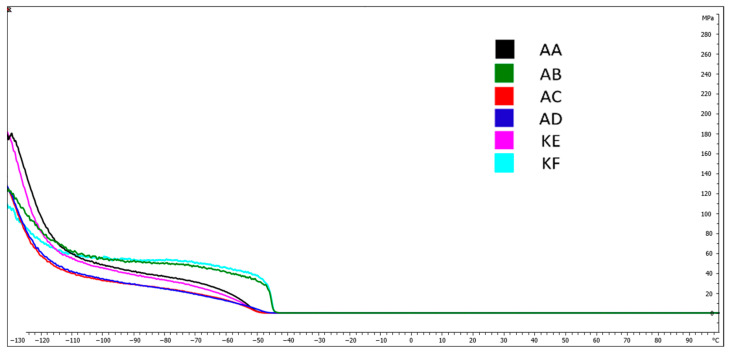
DMA curves−Loss modulus G″ for thermally aged samples.

Figure 9, Figure 10, Figure 11 and Figure 12 present DMA curves for the storage modulus G′ and the loss modulus G″. The DMA curves included in Figure 9 and Figure 11 correspond to reference samples while Figure 10 and Figure 12 correspond to samples after thermal aging. The analysis was conducted for repeatable testing parameters. The highest value of the curve for individual samples was determined at the second highest point on the curve. The initial values were considered as the material stabilization.

Performing a Dynamic Mechanical Analysis (DMA) showed a change in the storage modulus G′ of the thermally aged materials compared to the reference samples. An increase in the value of the G′ modulus was observed for the materials AD, KF, KE, and AA (Figure 10) while a 10% decrease occurred for the AC material. No change in the modulus value for the silicone AB was observed, which was within the range of 2100 MPa. The largest increase in value, amounting to 20%, occurred in the AD material.

Table 2 presents the temperatures for the highest points of the DMA curves for the storage and loss modulus. Based on the obtained results, no significant changes in the glass transition temperature values for the storage modulus were observed, which fluctuated within the range from 0.2 to 2.8%. Larger changes were observed in the loss modulus G″, where the change for the addition silicones AB and AD was 5.1% and 4.9%, respectively.

The DMA tests conducted did not reveal significant differences in the phase transition temperature into the elastic state of materials before and after subjecting them to high temperature (Table 3). The temperatures for the storage modulus oscillated around −37 to −43 °C while for the loss modulus, they were around −37 to −42.7 °C.

### 4.6. Fourier Transform Infrared Spectroscopy (FTIR)

**Figure 13:**
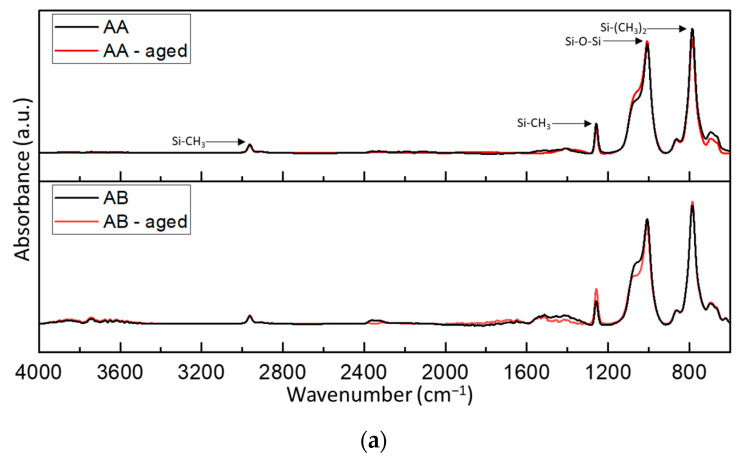

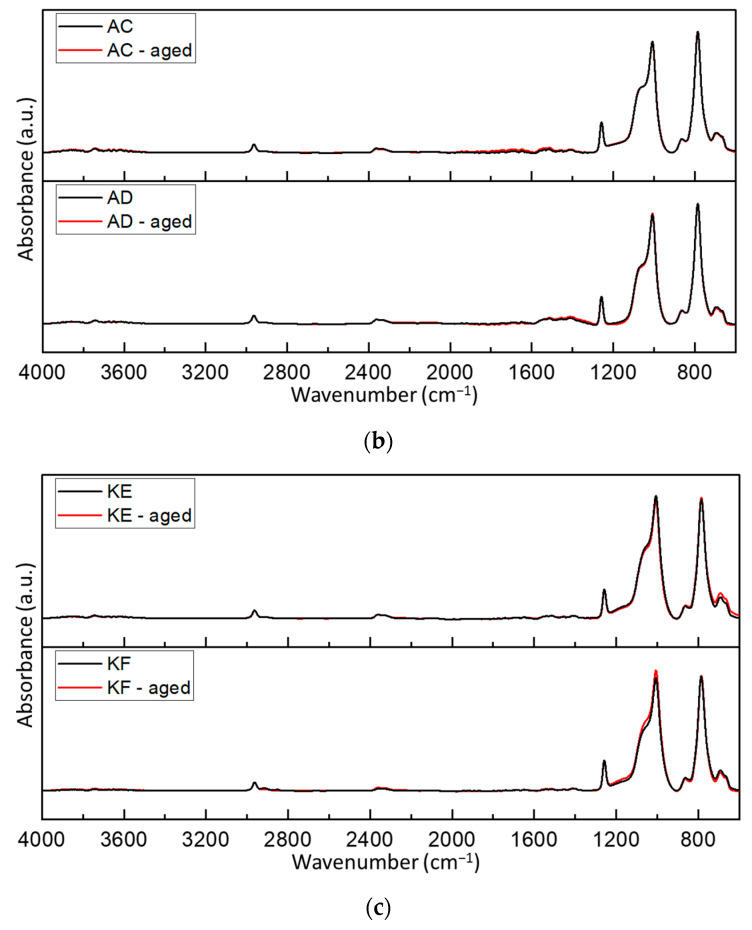
FTIR spectra of the reference and aged materials (**a**) AA and AB (**b**) AC and AD (**c**) KE and KF.

Figure 13 present the FTIR spectra for the tested materials. The analysis was conducted for both the samples before and after thermal aging. All tested materials exhibit peaks characteristic of poly(dimethylsiloxane) (PDMS) Si-O-Si, Si-CH_3_, and Si-(CH_3_)_2_).

The conducted FTIR spectroscopy indicates an increase in the intensity of the Si-O-Si band (1006 cm^−1^) for the AA material, and the Si-CH3 (2786 cm^−1^) and Si-(CH_3_)_2_ (800 cm^−1^) bands for the AB material (Figure 13a) after thermal aging. It is possible that the increase in the intensity of individual spectral bands can be associated with an increase in the mechanical properties of the materials after thermal aging, which was demonstrated earlier. In the remaining materials AC, AD, KE, and KF, significant changes in the intensity of individual bands were identified. For these materials, it is difficult to associate the demonstrated changes in material strength or their decrease with FTIR spectroscopy. 

The absence of significant differences in the FTIR spectra for the reference samples and those subjected to accelerated thermal aging suggests that there have been no significant changes in the chemical structure of the materials. No new peaks characteristic of potential degradation products of the tested PDMS were identified in any of the studies. This means that the changes observed in the individual studies likely correspond to the reorganization of polymer chains in the structure rather than changes in the chemical makeup of the materials. This indicates that any degradation mentioned in the analysis of roughness test results is superficial in nature and may only affect the surface layers of the materials.

## 5. Conclusions

Based on the results of the study, it was concluded:

- Thermal aging slightly affects the density of polyadditive and polycondensation silicones. A minor decrease in this value was observed. The change is minor and falls within the error margin.
- Thermal aging affects the change in Shore A hardness values. For polyadditive PDMS, there is a noticeable increase in the average hardness value, which is likely related to their long chains in the structure, in which further cross-linking of the structure occurs as a result of high temperature. Polycondensation PDMS show a decrease in average hardness values after thermal aging. This is likely due to a different structural build of the materials compared to polyadditive PDMS, where the chains are shorter and less resistant to external factors, such as a high temperature during which they break apart.
- The change in tensile strength results is conditioned by the type of material. In polyadditive PDMS, similar to the hardness results, the tensile strength increases and the greatest average increase in this value was observed for polyadditive silicones AC (3.4%) and AD (3.2%), which are characterized by higher initial hardness compared to other polyadditive silicones. Polycondensation silicones also show a change, but it is decreasing. In the case of polycondensation silicones, a greater average change in tensile strength value can be observed for the KF material (14.3%), which is characterized by a higher initial Shore A hardness value.
- Regardless of the type of material, a decrease in the average value of relative elongation is noticeable. From the obtained results, it can be concluded that the value of relative elongation in polyadditive PDMS depends on the viscosity of the material, as a much larger change in this value was observed for materials with lower viscosity. The greatest change in this value occurs for polyadditive PDMS materials AB (46.7%) and AC (49.7%).
- The effect of high temperature on the tested materials results in a minor change in surface roughness compared to reference materials. A comparable change occurs in both polyadditive and polycondensation PDMS.
- AC and AD materials, which are characterized by higher initial hardness (before thermal aging), have a higher storage modulus G’ than materials with lower hardness.
- For the polyadditive PDMS AA, an increase in the intensity of the Si-O-Si band was observed while for AB, there was an increase in the intensity of the Si-CH_3_ and Si(CH_3_)_2_ bands, which may be related to the increase in tensile strength or hardness results. For the remaining polyadditive silicones AC and AD and polycondensation silicones KE and KF, no significant change in the intensity of individual spectra is visible. It is not possible to unequivocally correlate the obtained spectra results with the results of mechanical tests.
- Essentially, it can be assumed that in the case of condensation PDMS, the observed changes are due to further cross-linking of the material under the influence of high temperature while for addition PDMS, stress relaxation also had a significant impact. The tested PDMS materials show satisfactory resistance to degradation at elevated temperatures. From a technical utilization standpoint of the tested materials, it can be stated that the observed changes will not have a significant impact on the durability and functionality of the products made from them. At the same time, it seems advisable to conduct tests on materials aged over a longer period.

## Figures and Tables

**Table 1:** Material properties based on manufacturer data [14,15,16,17,18,19].

Properties	Standards	Unit	AA	AB	AC	AD	KE	KF
Mixing proportion (base:catalyst)	-	-	100:10	100:100	100:100	100:10	100:5	100:5
Density 25 °C	ISO 1183 [20]	g/cm^3^	1.12	1.17	1.08	1.08	1.19	1.2
Brookfield viscosity	-	mPa s	20,000	3700	8000	35,000	25,000	35,000
Life time 25 °C	-	min	60	10–12	20–22	75	20–60	20–60 *
Demoulding time 23 °C	-	h	16	1	3	24	6	6

* dependent on the catalyst.

**Table 2:** Summary of the onset of glass transition temperature results for G′ and G″ curves.

Sample Type	Reference Sample [°C]		Aged Sample [°C]	
	Modulus G′ Peak	Modulus G″ Peak	Modulus G′ Peak	Modulus G″ Peak
AA	−112.17	−108.74	−111.95	−108.25
AB	−113.01	−108.42	−111.87	−102.91
AC	−112.88	−108.09	−109.75	−106.81
AD	−112.00	−105.39	−114.37	−110.59
KE	−112.39	−108.60	−110.69	−105.16
KF	−111.20	−106.76	−113.04	−105.37

**Table 3:** Summary of storage modulus G′ and loss modulus G″ results.

Sample Type	Reference Sample [°C]		Aged Sample [°C]	
	Modulus G′	Modulus G″	Modulus G′	Modulus G″
AA	−40.94	−42.05	−42.05	−41.08
AB	−43.27	−42.7	−42.70	−41.99
AC	−41.78	−40.41	−40.41	−40.10
AD	−40.19	−39.72	−39.72	−39.11
KE	−38.05	−37.06	−37.06	−37.02
KF	−38.21	−38.15	−38.15	−37.95

## Data Availability

Not applicable.

## Supplementary Materials

The following supporting information can be downloaded at: https://www.mdpi.com/article/10.3390/polym15193857/s1, Figure S1: Force−elongation curves (**a**) reference (**b**) aged samples of polyaddition silicone AB, Figure S2: Force-elongation curves (**a**) reference (**b**) aged samples of polycondensation silicone KE.
