# Supplementary material for: Analysis of Thermal Aging Influence on Selected Physical and Mechanical Characteristics of Polyaddition and Polycondensation Poly(dimethylsiloxane)
Source: Polymers (Basel). 2023 Sep 22;15(19):3857. doi: 10.3390/polym15193857 (PMC10574853; doi:10.3390/polym15193857)
Supplement: Supplementary file 1 [file polymers-15-03857-s001.zip › polymers-2580734-supplementary.pdf]

(a)

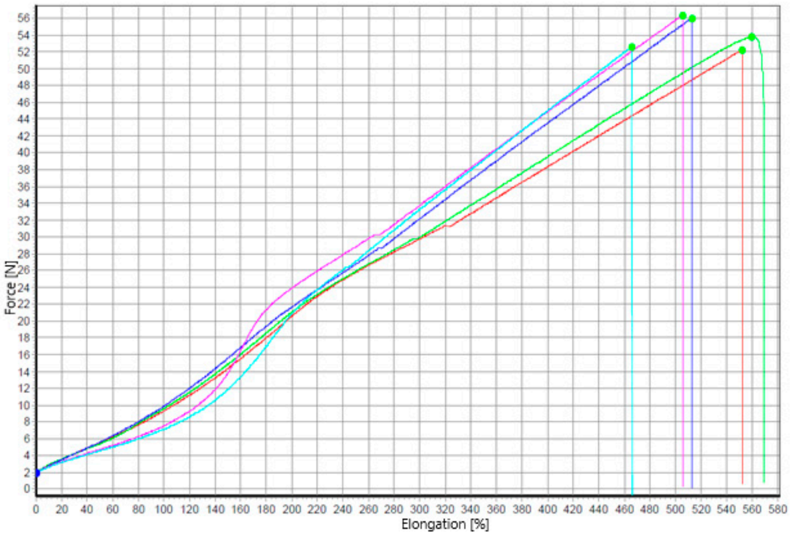

(b)

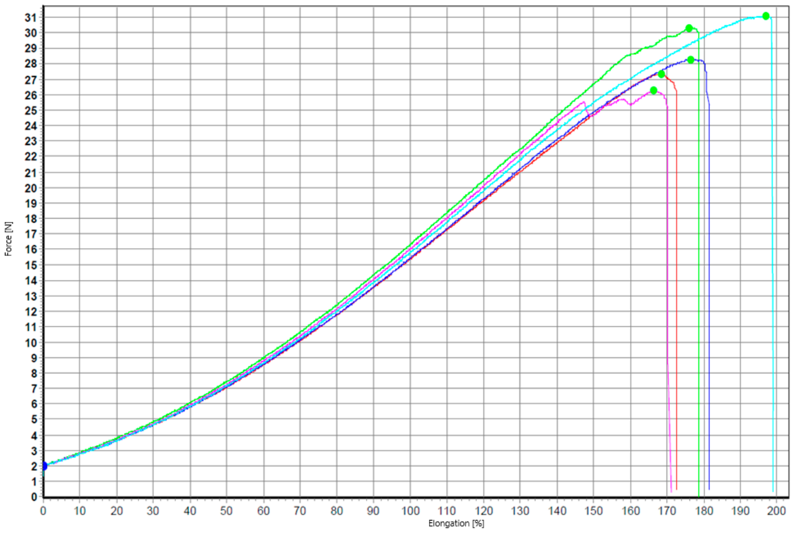

Figure S1. Force-elongation curves (**a**) reference (**F**) aged samples of polyaddition silicone AB.

(a)

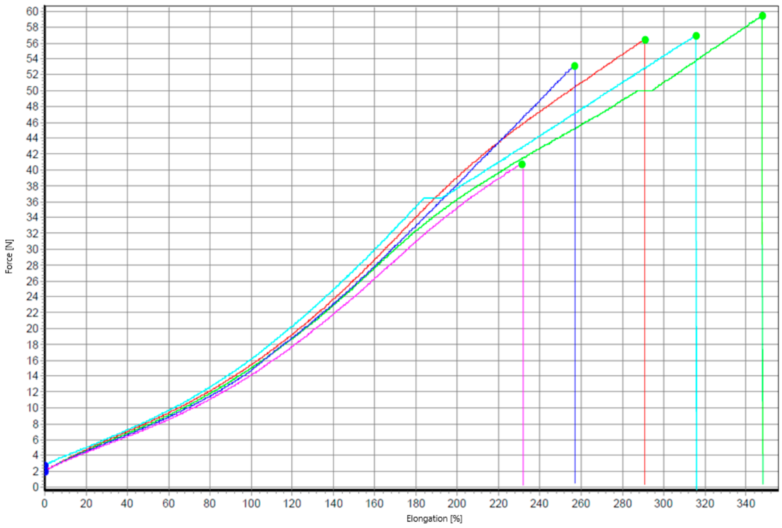

(b)

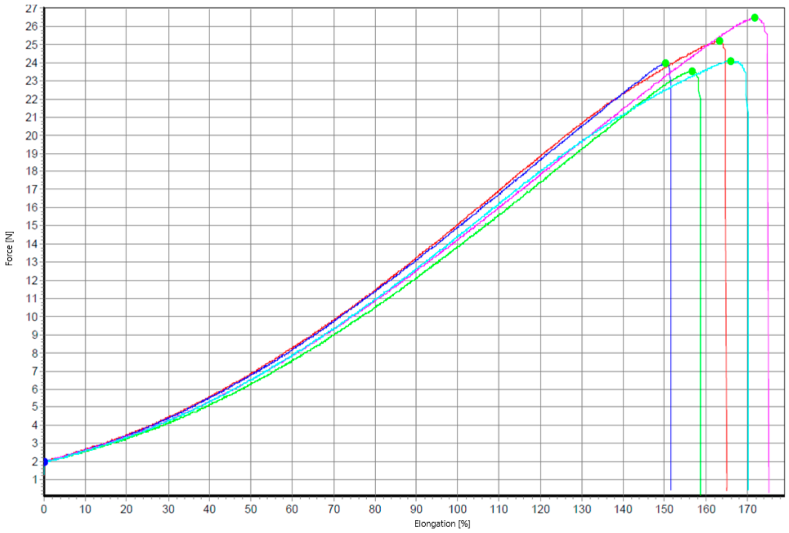

Figure S2. Force-elongation curves (**a**) reference (**b**) aged samples of polycondensation silicone KE.
